# Effects of dietary supplementation with different fermented feeds on performance, nutrient digestibility, and serum biochemical indexes of fattening lambs

**DOI:** 10.5713/ajas.20.0445

**Published:** 2020-08-30

**Authors:** Chen Zhang, Chongyu Zhang, Meiyu Du, Yunpeng Wang, Guiguo Zhang, Yunkyoung Lee

**Affiliations:** 1 College of Animal Sciences and Technology, Shandong Provincial Key Laboratory of Animal Biotechnology and Disease Control and Prevention, Shandong Agricultural University, Taian, Shandong 271018, China; 2 Department of Food Science and Nutrition, and Interdisciplinary Graduate Program in Advanced Convergence Technology and Science, Jeju National University, Jeju 63243, Korea

**Keywords:** Fermented Feedstuffs, Fatting Lambs, Growth Performance, Nutrient Digestibility

## Abstract

**Objective:**

The effects of adding fermented feed to a pelleted total mixed ration (PTMR) on the growth performance of lambs remain unclear. The present study aimed to investigate the feed efficiency and productivity of lambs that were fed PTMR containing fermented soybean meal (FSM) or wheat bran (FWB).

**Methods:**

Sixty 90-d-old hybrid lambs were randomly allocated into 12 pens (5 lambs/pen) that were randomly assigned to 4 dietary treatments (3 pens/treatment) with PTMR (basal diet), 2% FSM, or *Lactobacillus*- or yeast-FWB (L-FWB or Y-FWB) addition in the basal diet.

**Results:**

The findings showed that lambs fed 2% FSM supplemented diet had enhanced (p<0.05) average daily gain (ADG) and carcass yield (p = 0.015), while they had a decreased (p = 0.006) feed conversion ratio compared to that of other three groups. Inclusion of FSM or FWB in PTMR improved (p<0.05) the nutrient digestibility, while it reduced the urea nitrogen content in serum compared to the PTMR group. Additionally, the decreased ratio of N excretion to ADG (p<0.01) was observed with FSM and L-FWB supplementation compared with the PTMR and Y-FWB groups.

**Conclusion:**

In conclusion, feeding the fermented feed-supplemented diet improved nutrient digestibility and growth performance, and 2% FSM-supplemented diet exhibited superior production-promoting efficiency to lambs.

## INTRODUCTION

Soybean meal (SBM) and wheat bran are the most commonly applied by-products of grain processing in lambs’ feed products. However, many kinds of anti-nutritional factors (ANFs) such as trypsin inhibitors, soybean antigenic proteins, and phytic acid, were found in SBM and wheat bran, which have been shown to diminish the feed efficiency, disorder the gut microbiota, and cause intestinal inflammation and diarrhea, and thus decrease productivity and animal health status. Solid-state fermentation of feedstuffs by certain microorganisms has been extensively utilized to improve the nutrient quality by eliminating the ANFs and producing beneficial metabolites. It has been demonstrated that SBM and wheat bran fermented by suitable microbes play a positive role in enhancing feed efficiency and promoting the growth performance to piglets and broilers [1,2]. However, the nutritional quality of the fermented feed varied depending on the incubated strains and substrates. Bacteria (*Lactobacillus* sp.) and yeast (*Saccharomyces cerevisiae*) are commonly used in fermenting phytogenic feeds due to their capacity to eliminate ANFs and produce digestible enzymes such as protease, xylanase, and amylase [3]. Some previous studies have demonstrated the degradation of ANFs and increased small-sized peptides and amino acids by microbes in SBM- or wheat bran-based substrates [4–6]. Additionally, during the process, *Lactobacillus* can ferment water-soluble carbohydrates to produce organic acids, especially lactic acid [7]. Thus, fermentation is associated with a high number of lactic acid bacteria and a high concentration of organic acids besides the improved nutritional properties [8].

However, few studies concentrated on the effects of supplementing fermented feedstuffs in pelleted total mixed ration (PTMR) diet of lambs. The feed efficiency and impacts on productivities of feeding the fermented feedstuffs supplemented PTMR to fattening lambs remain unclear. The present studies aimed to evaluate i) the effects of feeding PTMR containing different fermented feedstuffs on fattening lambs’ production performance, and ii) the changes of nutrient digestibility, N excretion, and serum biochemical parameters by the fermented feedstuffs-supplemented PTMR on fattening lambs.

## MATERIALS AND METHODS

All lambs used in this study were cared for strictly following the animal care and use protocol that was approved by the Shandong Agricultural University Animal Nutrition Research Institute (Protocol No. 2019018).

### Preparation of the fermented feedstuffs

The fermented soybean meal (FSM) was produced as follows: 400 kg *Lactobacillus* solution (colony-forming unit [CFU], 3×10^9^/mL) was mixed with 600 kg substrate (soybean meal and starch, w:w = 9:1) in a spiral mixer with 2,000 kg capacity. After complete mixture, the materials were packed into a fermentation bag with a one-way vent valve and sealed for a 30-d fermentation. The *Lactobacillus*-fermented wheat bran (FWB) was fermented with the same procedure as with soybean meal but the substrate was composed of wheat bran and starch (w:w = 9:1) and was inoculated with yeast solution (CFU, 3×10^9^/mL). After a 30-d incubation, the fermented feed was used as a component formulated into the total mixed ration (TMR) diet.

### Experimental design, animals, diets, and feeding management

Sixty 90-d-old hybrid ram lambs (Australia Aries×Hu Sheep) with body weight (BW) of 22.5±0.5 kg were obtained from Shandong Agricultural University Research Farm (Shandong, China) and randomly allocated to 12 pens with 5 lambs in each pen, and pens were randomly assigned to 4 dietary treatments with 3 pens (replicates) per treatment. The experiment was conducted with a completely randomized experimental design and the pens were considered replicate units. The treatment groups were fed with basal only and basal diet added 2% FSM, wheat bran fermented with *Lactobacillus* (L-FWB), or yeast (Y-FWB) instead of soybean or wheat bran at the same proportion, respectively. Basal diets were formulated to meet the nutrient requirements recommended by the Feeding Standard of Meat-producing Sheep and Goats in China (NY/T 816-2004). The diet compositions and nutritional contents are shown in Table 1, and PTMR was prepared according to the methods described by Zhang et al [9]. Enough diets were produced in one batch to make sure there was no batch effect on dietary treatments. This experiment consisted of a 10-d adaptation period and an 84-d fattening period for sample collection. During the whole experimental period, all lambs had free access to the assigned diets and fresh tap water, and diets were offered two times a day (at 06:00 and 18:00 h).

The lambs were weighed on the ages of 100, 121, 163, and 184 d before morning feeding. Average daily gain (ADG) was calculated for d 100 to 121, 121 to 163, 163 to 184 by dividing the difference of measured weights by the period interval. The daily feed supply and orts for each pen were recorded to determine average daily feed intake (ADFI). The feed conversion ratio (FCR) was calculated by dividing ADFI by ADG.

### Determination of nutrient digestibility

Twenty 121-d-old healthy lambs (randomly selecting 5 lambs of each treatment) were housed individually in metabolism cages (130 cm in length, 100 cm in width and 150 cm in height) in a shed building to allow the total collection of feces and urine over 24 h. The digestion experiment was conducted for 9 d with the first 4 d as adaptation period and the remaining 5 d for sample collection. The daily feed offered, orts, and spillages were collected and weighed to determine ADFI. The subsample of each diet was taken daily at feeding, dried at 65°C, and ground using a 1.0-mm screen for chemical analysis. All feces and urine were individually collected immediately after excretion during the 5-d period. The daily excreta of each lamb collected at each time was weighted, and 10% H_2_SO_4_ was added at a ratio of 100 g of wet fecal sample to 10 mL 10% H_2_SO_4_, and subsequently stored in a sealed plastic bag at −20°C. At the same time, urine samples were collected in a bucket containing 1,000 mL of 10% H_2_SO_4_ to keep the final pH below 3 to prevent N losses. Every morning the collected urine was measured individually and to prevent the precipitation during storage and then stored at 4°C for the estimation. At the end of the 3-d period, all bags containing daily feces of each lamb were thawed at room temperature and mixed thoroughly. An equal amount of daily fecal sample from the same lamb was pooled and a single subsample (10% of the total weight) was then oven-dried at 65°C to constant weight and ground to pass a 1.0-mm screen for chemical analyses. On d 177, ten healthy lambs (5 lambs of each treatment) were used for the digestion experiment, which was conducted for 9 d with the first 4 d as adaptation period and the remaining 5 d for sample collection. Feed offered and orts of each lamb were recorded daily for determination of feed intake and feces were collected. The content of dry matter (DM), and crude protein (CP) of feeds, feces and urine were analyzed according to Association of Official Analytical Chemists (AOAC) [10]. The contents of neutral detergent fiber (NDF), and acid detergent fiber (ADF) were determined by the method of Van Soest et al [11]. Metabolizable energy was the calculated value from CSIRO [12].

### Sample collection and determination

Blood samples from all the animals were collected by jugular vein on d 120 and 170 of the fattening period just before morning feeding. Blood was collected in Li-heparin treated tubes that were centrifuged at 1,500×g for 20 min at 4°C, and separated plasma was stored at −20°C until further analysis. Total protein (TP), albumin, blood urea nitrogen (BUN), triglycerides (TG), and glucose (Glu) were measured using the automatic blood biochemical analyzer (7020, HITACHI, Tokyo, Japan).

On d 84 (184-d-old) of the fattening period and 12 h after the feeding, all the fatting lambs were taken and then slaughtered using electrically stunning according to the procedures recommended by the Animal Ethics Committee at Shandong Agricultural University, and subsequently the stunned lambs were bled within 20 s and then hung to remove their skin, head (at the occipital-atlantal joint), forefeet (at the carpal-metacarpal joint), and hind feet (at the tarsal-metatarsal joint) [13].

Carcass weight (kg) was measured after removing the total weight of the head, hoof, fur, blood, and viscus from the live weight before slaughter. Net meat weight (kg) was measured after removing all bones of the carcass. The carcass yield (%) was calculated by dividing the carcass weight by the live weight before slaughter. Net meat yield (%) was calculated by dividing neat meat weight by the carcass weight.

### Statistical analysis

In this study, a pen was the experimental unit for growth performance measurements (n = 3). Data for ADFI, ADG, and FCR were analyzed using MIXED procedure for repeated measures in the SAS version 9.0 (SAS Inst. Inc., Cary NC, USA) with treatment, period, and the interaction between treatment and period as fixed effects and each pen as a repeated measure, and the statistical model is as follows: *y_ij_* = *μ* +*T_i_* + *β*(*x_ij_* − *χ̄*) + *e_ij_*_,_ *y_ij_* = the observed dependent variable; μ = overall mean; *T_i_* = treatment effect; *x_ij_* = independent covariate; *β* = associated regression parameter; *β*(*x_ij_* − *χ̄*)= covariate effect; and *e_ij_* = experimental error. An autoregressive covariance was included in the model to adjust the time effect. BW, blood biochemical indexes, and slaughter performance were analyzed using one-way analysis of variance in the general linear model procedure. The significance of differences between the treatments was tested using LSMEANS with the PDIFF option. The difference was declared to be statistically significant when p<0.05.

## RESULTS

### Growth performance of lambs

**Figure 1:**
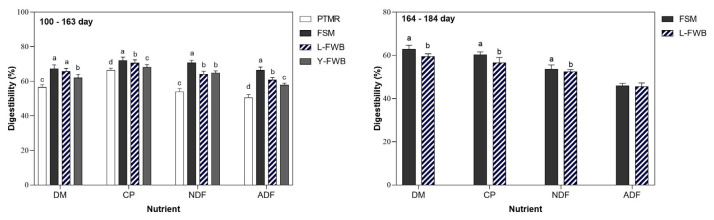
Effects of feeding PTMR containing different fermented feedstuffs on nutrient digestibility of lambs. PTMR, the pelleted total mixed ratio was the basal diet; FSM, basal diet supplemented with 2% fermented soybean meal; L-FWB, basal diet supplemented with 2% *Lactobacillus*-fermented wheat bran; Y-FWB, basal diet supplemented with 2% yeast-fermented wheat bran; DM, dry matter; CP, crude protein; NDF, neutral detergent fiber; ADF, acid detergent fiber.

The growth performances of lambs in different stages are presented in Table 2. Both PTMR and Y-FWB groups presented a decreased performance (ADG and FCR) and nutrient digestibility (Figure 1), while the nitrogen (N) excretion ratio increased (Table 3) from 100 to 163 d of age. Therefore, these two treatments were terminated on d 64 (164-d-old age) of the experiment.

No significant difference was observed for the initial weight among all groups. However, the lambs fed FSM-supplemented diet had improved (p<0.01) BW, ADG, whereas FCR decreased (p<0.01) among 4 experimental groups over the whole experimental stages. From 122 to 163 d of age, the lambs in the FSM and FWB groups had increased (p<0.01) BW, ADG, and ADFI, but reduced (p<0.01) FCR compared to the PTMR group, suggesting an enhanced feed efficiency and performance due to supplementation with fermented feeds in general. The enhanced (p<0.01) BW and lowered (p<0.01) FCR were observed by L-FWB supplemented group compared to the Y-FWB group showing higher growth-promoting efficiency of L-FWB than Y-FWB in lambs.

### Nutrient digestibility

The nutrient digestibility of lambs fed different fermented feedstuffs is shown in Figure 1. From 100 to 163 d of age, the lambs fed FSM- and FWB-supplemented diets had higher nutrient digestibility allowing for DM, CP, NDF, and ADF than the lambs fed PTMR diet (p<0.01). Meanwhile, an increased digestibility of CP, NDF, and ADF was observed by FSM supplemented group compared to the FWB-supplemented and PTMR groups. In addition, the L-FWB group had an enhanced (p<0.01) digestibility of DM, CP, NDF, and ADF compared with that of the Y-FWB group. Similarly, from 163 to 184 d, the lambs fed FSM-supplemented diet had a greater (p<0.05) digestibility of DM, CP, and NDF compared to the FWB-supplemented group.

### Nitrogen utilization

To estimate the effects of feeding fermented feedstuffs on N utilization, the N amounts of retention and excretion, the ratio of total N excretion to the ADG was calculated by determining the excretion of fecal and urinary N of 121- to 128-d-old lambs. Our findings indicated the N excretion ratio decreased (p = 0.016) and N retention ratio increased (p = 0.002), and that the ratio of total N excretion to ADG decreased (p<0.01) by FSM or L-FWB supplementation compared with that of Y-FWBY or PTMR groups. On the other hand, there was no significant difference in the ratio of N retention and excretion of FSM and L-FWB groups (Table 3).

### Serum biochemical indexes

In this study, the serum biochemical indexes were determined to evaluate the condition of nutrient metabolism and physiological activity of animals (Table 4). Supplementation of fermented feedstuffs in lambs’ diet improved (p<0.05) the concentration of TP, Glu, and TG, while it decreased the BUN content in serum suggesting the improvement of absorption and metabolism of protein and lipid in lambs. At 120 d-old of age, the lambs fed the FSM-supplemented diet had a greater globulin (Glo) and TG, while they had decreased BUN content in serum than that of lambs receiving FWB-supplemented diet. Similarly, higher contents of TP, Glo, and Glu were observed in the serum of the 183-d-old lambs receiving the FSM-supplemented diet than that fed L-FWB diet. On the other hand, concentrations of TG and BUN in the serum of the 183-d-old lambs were in the comparable range between FSM and L-FWB groups.

### Slaughtering performance

**Figure 2:**
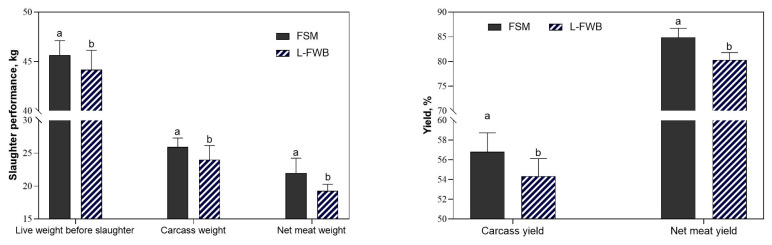
Effects of feeding pelleted total mixed ratio (PTMR) containing different fermented feeds on slaughter performance. FSM, basal diet supplemented with 2% fermented soybean meal; L-FWB, basal diet supplemented with 2% *Lactobacillus*-fermented wheat bran. ^a,b^ Means with different superscripts are different (p<0.05).

The BW and carcass weight at the end of the experimental period are shown in Figure 2. Lambs fed FSM supplemented diet had increased live BW before slaughter (p = 0.041), carcass weight (p = 0.030), net meat weight (p = 0.016), carcass yield (p = 0.015), and net carcass yield (p = 0.042) compared to the lambs fed L-FWB supplemented diet.

## DISCUSSION

It is important to develop an appropriate processing method to improve the feed utilization and to establish a stable TMR diet supply in intensive feedlot rearing of lambs [9,13]. The present findings displayed the supplementation of FSM or wheat bran in lambs’ PTMR diet can promote a better nutrient digestibility and growth performance compared with the non-supplementation group. Nutrient digestibility has a positive relationship with growth performance [14]. The results of this study demonstrated that supplementation of fermented feedstuffs to fatting lambs enhanced their growth performance in terms of ADG, which was consistent with previous studies. Yuan et al [2] documented that the fermented soybean can improve the growth performance, nutrient digestibility, and microbial flora in piglets. Zhang et al [15] demonstrated that a diet containing FSBM improved growth performance in piglets. Wang et al [16] found that feeding a diet containing 6% FSBM can result in greater growth performance in weanling pigs. The improved growth performance and similar ADFI suggested that increased growth performance was primarily due to increased nutrient digestibility. Similarly, Choi et al [17] found the fermented spent coffee grounds had a higher CP digestibility and N utilization in lambs. Luo et al [18] documented that of inclusion the FSM improved the antioxidant status and milk quality of ewes. Solid-status fermenting feedstuffs with beneficial microorganisms were considered as a feasible strategy for enhancing the nutritional quality by passivating the ANFs and producing the beneficial metabolites such as the organic acid, small peptides, and amino acids [3]. On the other hand, our previous studies have documented that feeding PTMR to fattening lambs can increase the abundance of beneficial bacteria and improve growth performance [9].

Additionally, the present findings indicated that supplementation of FSM and L-FWB reduced the N excretion with a lowered the ratio of Total N excretion to ADG. This indicated an improved ecological efficiency from the application of fermented feeds in lambs’ diet. Combining those results, adding the fermented feed into the lambs’ PTMR diet was beneficial to increasing nutrient utilization, improving performance, and obtaining a better ecological benefit. This provided a scientific reference for promoting PTMR and fermented feedstuffs in fattening lambs intensive raising system.

Another interesting finding in this study was the increased carcass yield and net meat yield by supplementation of FSM compared to the FWB group. This implied that the application of FSM in PTMR diet possibly regulated the nutrient biosynthesis and metabolism of muscle and lipid tissues in lambs which warrants further investigation. Similarly, Palma et al [19] addressed that a discrepant diet can affect the nutrient deposition in skeletal muscle by modifying the gastrointestinal microbiome and metabolome of sheep. Guo et al [20] found that partial substitution of FSM for soybean meal in broilers’ diet improved the carcass yield and meat quality of broiler chickens. Thus, types of fermented feed can differentially impact the growth performance and meat quality of lambs.

The supplementation of fermented feedstuffs exhibited a beneficial impact on growth performance, while feed efficiency was tightly linked to the species of incubated strains and fermented substrate. In this study, the *Lactobacillus*-FSM (FSM group) exhibited a superior growth-promoting function than L-FWB or Y-FWB, and PTMR diet, while the feeding efficiency of L-FWB group was higher than that of the Y-FWB group. This may be related to the fact that the *Lactobacillus*-fermented feed contributed to the colonization of the growth-promoting microbial community in the rumen, which further improved the ruminal fermentation efficiency and the nutrient digestibility. Zhang et al [21] found the oral administration of the probiotics affected the rumen bacterial community and the numbers of cellulolytic bacteria decreased. Lettat et al [22] demonstrated that *Lactobacillus* probiotic strains may be effective in stabilizing ruminal pH and therefore preventing acidosis risk. Zhang et al [23] reported that inoculating LAB in alfalfa silages can decrease pH, increase the production of lactic and acetic acids, reduce the number of yeasts and molds, and inhibit *Enterobacterium* and *K. pneumoniae*.

The BUN can reflect the metabolism of protein and the balance of dietary amino acids, and thus serves as an indicator of the N utilization efficiency [24,25]. It has been found that BW gain of lambs with high level had a positive correlation with total protein and Glo [26]. In this study, the lambs fed FSM- and L-FWB-supplemented diet had a decreased level of BUN while they had an increased content of serum total protein and Glo, which suggested an enhancedment of the dietary protein bioavailability and improved immune condition of the lambs due to the supplementation of fermented feeds [24]. Yeh et al [27] found that pelleted fermented feed improved broiler growth performance, which may be due to the higher digestible amino acid content. Consistent results were reported that lambs in the improved growth performance group had a statistically lower concentration of BUN [28] and a higher concentration of Glu [29].

It is needed to point out that there are some amounts of organic acids (acetic, propionic, and butyric acid, etc.) and beneficial bacteria in the fermented feedstuffs, which have beneficial impacts on animals’ gut microbiota, growth, and health status. Thus, further studies are worthy to investigate the underlying mechanisms of fermented feeds promoting the animals’ production performance.

## CONCLUSION

In conclusion, inclusion of fermented feedstuffs in the diet for fattening lambs improved the nutrient digestibility and health status, which consequently contributed to the superior growth and slaughtering performances, as well as the reduced N excretion. In this study, supplementation with 2% FSM in the fattening lambs’ diet displayed the optimal productivity and ecological efficiency. Thus, it is a feasible strategy to improve the performance of fattening lambs by adding FSM in the diet. This finding provided a scientific reference for applying fermented feed in the intensive feed-lot style feeding system of lambs.

## Figures and Tables

**Table 1:** Composition and nutrient levels of experimental diets (%, DM basis)

Items	Treatments1)			

	PTMR	FSM	L-FWB	Y-FWB
Ingredients				
Peanut vine	30	30	30	30
Corn	24	24	24	24
Soybean meal	15	13	15	15
wheat middlings	14.4	14.4	14.4	14.4
Leymus chinensis	13	13	13	13
FSM	0	2	0	0
L-FWB	0	0	2	0
Y-FWB	0	0	0	2
Wheat bran	2	2	0	0
CaHPO_4_	0.44	0.44	0.44	0.44
Limestone	0.4	0.4	0.4	0.4
Sodium chloride	0.4	0.4	0.4	0.4
Premix2)	0.36	0.36	0.36	0.36
Total	100	100	100	100
Nutrient content				
Dry matter (%)	90.84	90.18	89.48	90.6
Metabolizable energy3) (MJ/kg)	9.67	9.75	9.71	9.79
Crude protein (%)	14.33	14.94	14.49	14.42
Neutral detergent fiber (%)	32.44	31.55	31.88	31.07
Acid detergent fiber (%)	19.94	19.91	19.84	19.03
Calcium (%)	0.61	0.66	0.67	0.68

1) PTMR, the pelleted total mixed ratio was the basal diet; FSM, basal diet supplemented 2% fermented soybean meal; L-FWB, basal diet supplemented with 2% *Lactobacillus*-fermented wheat bran; Y-FWB, basal diet supplemented with 2% yeast-fermented wheat bran.

2) Supplied per kg of total mixed ration: vitamin A 1,367 IU, vitamin D 194 IU, vitamin E 15 IU, Fe (FeSO_4_·7H_2_O) 74 mg, Zn (ZnSO_4_·7H_2_O) 46.3 mg, Mn (MnSO_4_·5H_2_O) 36.5 mg, Cu (CuSO_4_·5H_2_O) 17.0 mg, I (KI) 1.5 mg, Co (CoCl_2_·6H_2_O) 0.3 mg, Se (Na_2_SeO_3_) 0.3 mg.

3) ME was calculated following CSIRO method, ME = 0.134 DMD+0.235EE+1.23, ME, metabolizable energy; DMD, dry matter digestibility; EE, ether extract [12].

**Table 2:** Effects of feeding pelleted total mixed ratio containing different fermented feedstuffs on production performances of lambs

Items	Treatments1)				SEM	p-value

	PTMR	FSM	L-FWB	Y-FWB		
BW (kg)						
100 d of age	22.63	22.65	22.61	22.58	0.162	0.853
121 d of age	27.16^bc^	27.63^a^	27.41^ab^	26.92^c^	0.16	0.014
163 d of age	37.00^d^	39.26^a^	38.58^b^	38.18^c^	0.406	0.001
184 d of age	-	45.31^a^	44.10^b^	-	0.402	0.010
100 to 121 d of age						
ADG (g/d)	215^c^	237^a^	228^b^	206^d^	1.092	0.003
ADFI (g/d)	995	992	992	983	2.973	0.235
FCR	4.61^b^	4.18^d^	4.34^c^	4.76^a^	0.021	0.003
122 to163 d of age						
ADG (g/d)	234^c^	277^a^	266^b^	268^b^	0.7	<0.001
ADFI (g/d)	1,391^c^	1,453^b^	1,428^b^	1,514^a^	4.325	<0.001
FCR	5.94^a^	5.24^d^	5.37^c^	5.64^b^	0.02	<0.001
164 to 184 d of age						
ADG (g/d)	-	288^a^	263^b^	-	9.802	0.005
ADFI (g/d)	-	1,802	1,730	-	10.582	0.280
FCR	-	6.26^b^	6.58^a^	-	0.283	0.030
Whole period (100 to 184 d of age)						
ADG (g/d)	-	269^a^	256^b^	-	5.153	0.013
ADFI (g/d)	-	1,330	1,310	-	9.576	0.151
FCR	-	4.93^b^	5.12^a^	-	0.127	0.006

SEM, standard error of the mean; BW, body weight; ADG, average daily gain; ADFI, average daily feed intake; FCR, feed conversion ratio.

1) PTMR, the pelleted total mixed ratio (PTMR) was the basal diet; FSM, basal diet supplemented with 2% fermented soybean meal; L-FWB, basal diet supplemented with 2% *Lactobacillus*-fermented wheat bran; Y-FWB, basal diet supplemented with 2% yeast-fermented wheat bran. The PTMR and Y-FWB groups were terminated for the poor performance on d 64 (164-d-old age) of the experiment.

a–d Means within a row with different superscript letters are different (p<0.05).

**Table 3:** Effects of feeding pelleted total mixed ratio containing different fermented feedstuffs on nitrogen (N) utilization of lambs

Items	Treatments1)				SEM	p-value

	PTMR	FSM	L-FWBL	Y-FWB		
Nitrogen intake (g/d)	26.84	27.82	26.42	28.69	1.131	0.012
Excretion in feces (g/d)	7.93^b^	7.69^c^	7.30^c^	8.21^a^	0.042	0.003
Excretion in urine (g/d)	8.68^b^	8.61^b^	8.51^b^	9.28^a^	0.04	<0.001
Total excretion (g/d)	16.61^b^	16.30^b^	15.81^c^	17.49^a^	0.376	0.011
N excretion ratio (%)	61.89^a^	58.59^b^	59.84^b^	60.96^a^	2.381	0.016
T-excretion/ADG (g/g)	0.077^b^	0.073^c^	0.069^c^	0.081^a^	0.01	0.001
N retention amount (g/d)	10.23^b^	11.52^a^	10.62^b^	11.19^a^	0.121	0.028
Nitrogen retention ratio (%)	38.11^b^	41.41^a^	40.17^a^	39.02^b^	0.27	0.002

SEM, standard error of the mean; ADG, average daily gain.

1) P TMR, the pelleted total mixed ratio (PTMR) was the basal diet; FSM, basal diet supplemented with 2% fermented soybean meal; L-FWB, basal diet supplemented with 2% *Lactobacillus*-fermented wheat bran; Y-FWB, basal diet supplemented with 2% yeast-fermented wheat bran.

a–c Means within a row with different superscript letters are different (p<0.05).

**Table 4:** Effects of feeding pelleted total mixed ratio containing different fermented feedstuffs on serum biochemical parameters of lambs

Items	Treatments1)				SEM	p-value

	PTMR	FSM	L-FWB	Y-FWB		
120 d of age						
Total protein (g/L)	56.90^c^	62.40^a^	59.40^b^	62.87^a^	0.26	<0.001
Globulin (g/L)	31.75^c^	39.87^a^	31.35^c^	34.15^b^	0.261	<0.001
Albumin (g/L)	25.15^b^	22.53^c^	28.05^a^	28.72^a^	0.141	<0.001
Glucose (mmol/L)	3.46^b^	4.69^a^	4.82^a^	4.80^a^	0.036	<0.001
Triglyceride (mmol/L)	0.23^d^	0.41^a^	0.27^c^	0.35^b^	0.007	<0.001
Blood urea-N (mmol/L)	6.13^a^	4.26^c^	5.25^b^	6.35^a^	0.047	<0.001
170 d of age						
Total protein (g/L)	-	67.67^a^	66.43^b^	-	0.56	0.034
Globulin (g/L)	-	38.53^a^	37.10^b^	-	0.372	0.008
Albumin (g/L)	-	29.13	29.33	-	0.272	0.79
Glucose (mmol/L)	-	5.01^a^	4.30^b^	-	0.03	<0.001
Triglyceride (mmol/L)	-	0.43	0.45	-	0.113	0.319
Blood urea-N (mmol/L)	-	5.19	5.41	-	0.082	0.112

SEM, standard error of the mean.

1) PTMR, the pelleted total mixed ratio (PTMR) was the basal diet; FSM, basal diet supplemented with 2% fermented soybean meal; L-FWB, basal diet supplemented with 2% *Lactobacillus*-fermented wheat bran; Y-FWB, basal diet supplemented with 2% yeast-fermented wheat bran.

a–d Means within a row with different superscript letters are different (p<0.05).
